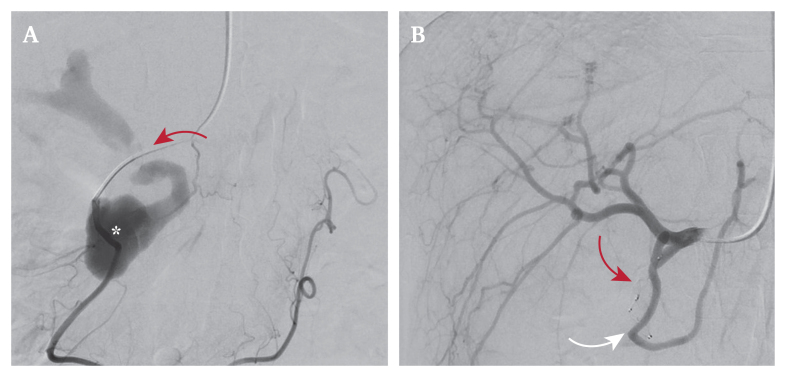# Transarterial Embolisation of a Gastroduodenal Artery Pseudoaneurysm Rupture Into the Portal Vein

**DOI:** 10.1016/j.ejvsvf.2023.10.001

**Published:** 2023-10-27

**Authors:** Hunor Sarkadi, Csaba Csobay-Novák

**Affiliations:** Department of Interventional Radiology, Semmelweis University, Budapest, Hungary

A 34 year old female presented with upper abdominal pain and nausea. Her medical history included alcoholic chronic pancreatitis. Computed tomography angiography revealed a gastroduodenal artery pseudoaneurysm with a portal fistula associated with chronic pancreatitis. Angiography (Fig. A) showed an eccentric sac measuring 26 x 38 mm (asterisk) with a small fistula to the portal vein (red arrow). Front (8 mm; Fig. B, red arrow) and back door (6 mm; Fig. B, white arrow) embolisation was performed using Amplatzer Vascular Plugs (AVP4, Abbott Park, Illinois, USA), excluding the pseudoaneurysm while preserving the anterior pancreaticoduodenal artery and common hepatic artery arising from the superior mesenteric artery. The patient was asymptomatic one month after the procedure.

**Figure:**